# Successful Re-administration of Granulocyte Colony-Stimulating Factor Without Recurrence of Associated Vasculitis: A Case Report

**DOI:** 10.7759/cureus.95211

**Published:** 2025-10-23

**Authors:** Kazuaki Teshima, Satoshi Takahashi, Shodai Takahashi, Nanako Fujita, Masaaki Kume, Naoto Takahashi

**Affiliations:** 1 Department of Hematology, Hiraka General Hospital, Yokote, JPN; 2 Department of Radiology, Hiraka General Hospital, Yokote, JPN; 3 Department of Hematology, Nephrology and Rheumatology, Akita University Graduate School of Medicine, Akita, JPN

**Keywords:** diffuse large b-cell lymphoma, filgrastim, granulocyte colony-stimulating factor, pegfilgrastim, re-administration, vasculitis

## Abstract

We report a case of diffuse large B-cell lymphoma complicated by granulocyte colony-stimulating factor (G-CSF)-associated vasculitis following filgrastim administration. The vasculitis resolved with corticosteroid therapy, and maintenance treatment was continued long-term. During salvage chemotherapy for lymphoma relapse 14 months after the initial episode, G-CSF was reintroduced because of persistent myelosuppression. Filgrastim and pegfilgrastim were used in subsequent cycles without recurrence of vasculitis. This case suggests that G-CSF re-administration may be cautiously considered on an individual basis, particularly when vasculitis is well controlled with corticosteroids and a sufficient interval has elapsed. These findings may help guide individualized management in similar clinical situations.

## Introduction

Granulocyte colony-stimulating factor (G-CSF) is widely used to reduce the risk of febrile neutropenia during chemotherapy. However, G-CSF-associated vasculitis, a form of large-vessel inflammation, has been reported with various formulations of G-CSF, including filgrastim and pegfilgrastim [1-4]. Management typically involves discontinuation of G-CSF and initiation of corticosteroid therapy when necessary [1-4]. Recurrence of vasculitis after G-CSF re-administration has been described; however, optimal management strategies remain unclear [5-10]. Here, we report a case of successful G-CSF re-administration without recurrence of G-CSF-associated vasculitis and discuss related clinical considerations.

## Case presentation

A 78-year-old woman presented with enlarged tonsils and was diagnosed with stage IIA diffuse large B-cell lymphoma (DLBCL) without B symptoms. The patient received first-line chemotherapy with R-CHOP (rituximab, cyclophosphamide, doxorubicin, vincristine, and prednisolone). On Day 12 of the first treatment cycle, the patient developed febrile neutropenia and was administered filgrastim (75 µg) and antibiotics. Filgrastim was given for seven consecutive days starting on Day 12; although the fever temporarily resolved, it recurred on Day 19 in conjunction with cervical pain. Laboratory tests showed marked inflammation, with a white blood cell count of 25,400/µL, erythrocyte sedimentation rate of 126 mm/h, and C-reactive protein level of 20.6 mg/dL (Table 1). Autoimmune serologic testing, including antinuclear antibodies, myeloperoxidase-antineutrophil cytoplasmic antibodies (ANCA), and proteinase 3-ANCA, yielded negative results (Table 1). The serum procalcitonin level was within the normal range, and blood cultures were negative, indicating no evidence of infection.

**Table 1:** Laboratory findings at the onset of vasculitis.

Test item	Result	Reference range	Unit
White blood cell count (WBC)	25,400	3,300–8,600	/µL
Segmented neutrophil	89	38–74	%
Lymphocyte	6	16–49	%
Monocyte	5	2–10	%
Hemoglobin (Hb)	12.7	11.6–14.8	g/dL
Platelet count (PLT)	196,000	158,000–348,000	/µL
Sodium (Na)	136	138–145	mmol/L
Potassium (K)	3.9	3.6–4.8	mmol/L
Chloride (Cl)	100	101–108	mmol/L
Calcium (Ca)	8.7	8.8–10.1	mg/dL
Blood urea nitrogen (BUN)	7.1	8–20	mg/dL
Creatinine (Cr)	0.68	0.46–0.79	mg/dL
Total bilirubin (T-Bil)	0.8	0.4–1.5	mg/dL
Aspartate aminotransferase (AST)	28	13–30	U/L
Alanine aminotransferase (ALT)	40	7–23	U/L
Lactate dehydrogenase (LDH)	163	124–222	U/L
Alkaline phosphatase (ALP)	198	38–113	U/L
γ-Glutamyl transferase (γ-GTP)	322	9–32	U/L
C-reactive protein (CRP)	20.6	0–0.14	mg/dL
Procalcitonin	0.27	0–0.49	ng/mL
Erythrocyte sedimentation rate (ESR)	126	3–15	mm/hr
Soluble interleukin-2 receptor (sIL-2R)	1,409	122–496	U/mL
Antinuclear antibody (ANA)	<40	<40	–
Myeloperoxidase-ANCA (MPO-ANCA)	Negative	Negative	–
Proteinase 3-ANCA (PR3-ANCA)	Negative	Negative	–
IgG	943	861–1,747	mg/dL
IgA	293	93–393	mg/dL
IgM	23	50–269	mg/dL
Complement C3 (C3)	164	73–138	mg/dL
Complement C4 (C4)	39	11–31	mg/dL
Total hemolytic complement (CH50)	86	30–45	U/mL
Blood glucose	102	70–109	mg/dL

**Figure 1:**
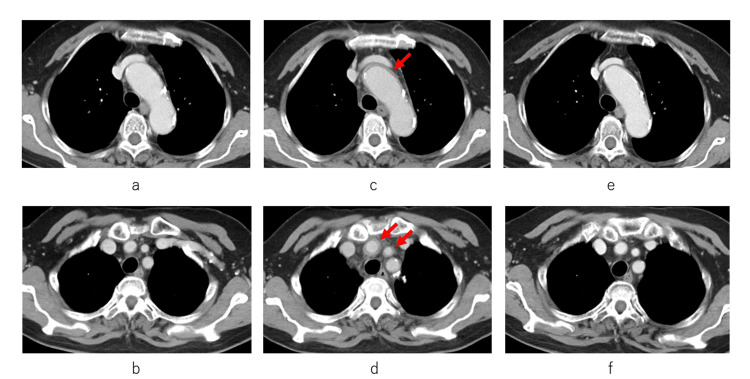
Serial contrast-enhanced computed tomography (CT) images showing changes in the aortic arch and supra-aortic vessels. (a, c, e) Aortic arch; (b, d, f) Brachiocephalic and common carotid arteries. (a, b) Baseline CT before the initial administration of filgrastim. (c, d) CT performed 8 days after initial filgrastim administration, depicting wall thickening consistent with vasculitis (arrows). (e, f) CT obtained 2 months after the onset of vasculitis, demonstrating an improvement in vascular inflammation.

Contrast-enhanced computed tomography (CT) revealed circumferential wall thickening of the aortic arch compared with previous imaging (Figure 1a-d). After excluding infections and autoimmune diseases, G-CSF-associated vasculitis was suspected based on previous reports of this rare condition [1-4]. The patient was administered methylprednisolone (500 mg) daily for 3 days, followed by oral prednisolone (40 mg, corresponding to 1 mg/kg). The oral prednisolone dose was subsequently tapered over 6 months, initially by 5 mg every 1-2 weeks until the dose reached 20 mg, and thereafter by 1-2.5 mg every 1-2 weeks, until complete discontinuation. G-CSF was discontinued, and subsequent R-CHOP cycles were administered with dose reductions. Two months later, CT revealed alleviation in vascular inflammation (Figure 1e, f). The patient completed six cycles and achieved complete lymphoma remission.

**Figure 2:**
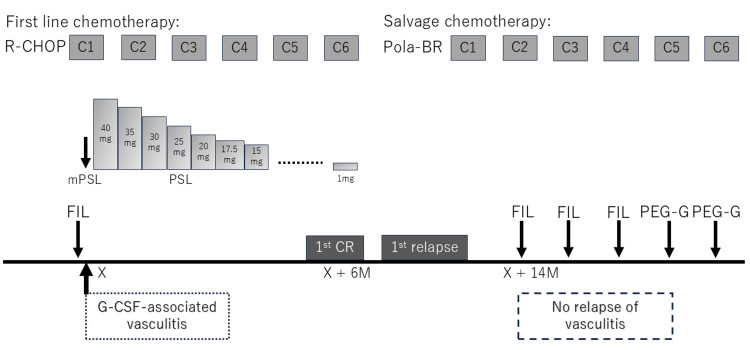
Temporal relationship between G-CSF use and the development or absence of vasculitis. The patient developed G-CSF-associated vasculitis following the administration of filgrastim during the first cycle of initial R-CHOP (rituximab, cyclophosphamide, doxorubicin, vincristine, and prednisolone) chemotherapy. After three days of intravenous methylprednisolone therapy, oral prednisolone was initiated at an initial dose of 40 mg. Subsequently, oral prednisolone was gradually tapered, being discontinued over six months. Filgrastim was subsequently discontinued during the first-line chemotherapy. Complete remission was achieved after six cycles of R-CHOP. At the time of the first relapse, salvage chemotherapy with polatuzumab vedotin, bendamustine, and rituximab (Pola-BR) was initiated. Filgrastim was administered during cycles 2 to 4 and pegfilgrastim during cycles 5 and 6. Vasculitis did not recur following G-CSF re-administration. The interval between the onset of G-CSF-associated vasculitis and re-administration of filgrastim was 14 months. CR, complete remission; FIL, filgrastim; mPSL, methylprednisolone; PEG-G, pegfilgrastim; PSL, prednisolone; M, months.

Seven months later, the patient experienced a relapse of cervical lymphadenopathy. A biopsy confirmed DLBCL recurrence. Salvage chemotherapy consisting of polatuzumab vedotin, bendamustine, and rituximab (Pola-BR) was initiated. During the first cycle, the patient developed grade 4 myelosuppression. Owing to prior vasculitis, G-CSF was initially withheld; however, persistent cytopenia led to re-administration of filgrastim (75 µg), beginning in the second cycle (14 months after the initial episode of vasculitis), after obtaining comprehensive informed consent. From the second to the fourth cycle, she received filgrastim 6-7 times per cycle without any recurrence of vasculitis. In the fourth cycle, febrile neutropenia occurred, prompting the use of pegfilgrastim (3.6 mg) in cycles 5 and 6, with administration performed on day 5 in both cycles. No recurrence of vasculitis was observed. The patient completed salvage therapy without dose reduction and remained in lymphoma remission for 18 months. The successful clinical course is shown in Figure 2.

## Discussion

G-CSF-associated vasculitis was initially described by Darie et al. [11]. Since then, numerous cases have been reported, particularly among Japanese women [10]. This condition, characterized by features of large-vessel vasculitis, typically affects individuals over 50 years of age, with a female predominance [12]. It is associated with elevated levels of inflammatory markers and imaging findings of perivascular soft tissue thickening, particularly involving the aortic arch and its major branches [12]. Traditionally, large-vessel vasculitis has been classified as Takayasu arteritis or giant cell arteritis [13]. Takayasu arteritis predominantly affects young women, whereas giant cell arteritis typically occurs in older women and is often associated with symptoms such as new-onset headache and temporal artery tenderness [14, 15]. In the present case, the patient’s age at onset was inconsistent with the demographic profile of Takayasu arteritis, and no clinical signs suggestive of giant cell arteritis were observed.

Before concluding that the vasculitis in this case was G-CSF-induced, we also considered other possible etiologies, including chemotherapy-related and lymphoma-associated vasculitis. Taimen et al. reported a case series and systematic review describing G-CSF- and chemotherapy-induced large-vessel vasculitis, all of which occurred in patients with breast cancer receiving these agents concomitantly [16]. These findings suggest that chemotherapy itself might contribute to vascular inflammation in certain contexts. However, reports on R-CHOP-induced large-vessel vasculitis are extremely limited, suggesting a low likelihood of direct causality in our case. Ito et al. further described a patient with DLBCL who developed vasculitis after pegfilgrastim administration during R-CHOP therapy [6]. Although chemotherapy was ongoing, the authors attributed vasculitis development primarily to G-CSF exposure.

Paraneoplastic vasculitis related to malignant lymphoma has also been documented. However, most such cases involved small-vessel vasculitis affecting the skin [17], differing from the clinical and radiologic pattern of large-vessel vasculitis in our case. Moreover, infectious and autoimmune etiologies were carefully ruled out in our patient. Taken together with the close temporal relationship between G-CSF administration and vasculitis onset, these findings support a drug-induced mechanism as the most plausible explanation. Although paraneoplastic vasculitis can occur in patients with malignancies, it usually arises independently of pharmacologic exposure [18]. However, the close temporal relationship between G-CSF administration and vasculitis onset in this case supports a drug-induced etiology.

G-CSF promotes the proliferation, mobilization, and activation of neutrophils, which can lead to the release of inflammatory cytokines such as interleukin-6 and tumor necrosis factor-α [19, 20]. Moreover, G-CSF may stimulate cytokine production by Th17 cells, amplifying the inflammatory cascade [19, 20]. These mechanisms may contribute to vasculitis through neutrophil priming and cytokine-mediated endothelial damage.

The optimal approach for the re-administration of G-CSF after prior vasculitis remains unclear. Among the published cases, recurrence was observed in six patients after G-CSF rechallenge, even when the formulation was changed [5-10] (Table 2). Only one previous case reported successful re-administration without recurrence of vasculitis after switching from pegfilgrastim to filgrastim [21]. Our case is unique in that it demonstrates that filgrastim and pegfilgrastim can be safely reintroduced.

**Table 2:** Summary of previously reported cases of G-CSF re-administration. F, female; M, male; BC, breast cancer; DLBCL, diffuse large B-cell lymphoma; OC, ovarian cancer; NEC, neuroendocrine carcinoma; PC, pancreatic cancer; PEG-G, pegfilgrastim; FIL, filgrastim;  Re-adm, G-CSF at the re-administration; NR, not reported; M, months.

Case	Age	Sex	Disease	G-CSF	Re-adm	Steroid use at initial episode	Interval	Recurrence	Reference
1	45	F	BC	PEG-G	FIL	Yes	NR	Yes	Lee et al. [5]
2	59	F	DLBCL	PEG-G	PEG-G	No	1 M	Yes	Ito et al. [6]
3	62	F	DLBCL	PEG-G	PEG-G	No	1 M	Yes	Sasaki et al. [7]
4	71	F	OC	PEG-G	PEG-G	No	3 M	Yes	Kawahara et al. [8]
5	71	M	NEC	PEG-G	FIL	Yes	8 M	Yes	Seto et al. [9]
6	65	F	PC	PEG-G	PEG-G	No	4 M	Yes	Shirai et al. [10]
7	61	F	BC	PEG-G	FIL	No	2 M	No	Chino et al. [21]
Present	78	F	DLBCL	FIL	FIL/PEG-G	Yes	14 M	No	

Two factors may have contributed to this favorable outcome: (1) prolonged corticosteroid therapy at initial presentation (approximately six months), potentially suppressing cytokine-driven inflammation; and (2) the long interval (approximately 14 months) between the initial episode and re-administration. Among reported cases of G-CSF rechallenge with recurrence of vasculitis, corticosteroids were withheld during the initial episode of vasculitis in all but two patients [5-10] (Table 2). Unexpectedly, recurrence occurred in one patient treated with steroids, with a relatively long interval before re-administration [9]. Thus, other factors such as steroid dosage, duration, and individual susceptibility might also affect the risk of recurrence.

Our findings indicate that re-administration of G-CSF should be carefully evaluated in each patient, particularly when vasculitis is initially well controlled with corticosteroids and a sufficient period has elapsed. Given the essential role of G-CSF in preventing febrile neutropenia and maintaining chemotherapy dose intensity, this case supports the careful consideration of G-CSF use in patients with a history of G-CSF-associated vasculitis.

## Conclusions

In a patient with DLBCL who previously developed G-CSF-associated vasculitis, re-administration of filgrastim and pegfilgrastim without recurrence was deemed feasible after achieving durable control of inflammation with corticosteroids and a 14-month interval. This approach can be considered with caution on an individual basis, accompanied by careful monitoring and shared decision-making after G-CSF re-administration. Further studies are required to define standardized criteria to guide the safe re-administration of G-CSF in this setting.
